# Fabrication of buried nanostructures by atomic layer deposition

**DOI:** 10.1038/s41598-018-33036-3

**Published:** 2018-10-10

**Authors:** Rizwan Ali, Muhammad Rizwan Saleem, Matthieu Roussey, Jari Turunen, Seppo Honkanen

**Affiliations:** 1 Institute of Photonics, University of Eastern Finland, Joensuu, P.O. Box 111, FI-80101 Finland; 2 U.S.-Pakistan Center for Advanced Studies in Energy (USPCAS-E), National University of Sciences and Technology (NUST), Sector H-12, Islamabad, P.O. Box 44000, Pakistan

## Abstract

We present a method for fabricating buried nanostructures by growing a dielectric cover layer on a corrugated surface profile by atomic layer deposition of TiO_2_. Selecting appropriate process parameters, the conformal growth of TiO_2_ results in a smooth, nearly flat-top surface of the structure. Such a hard surface can be easily cleaned without damage, making the nanostructure reusable after contamination. The technique has wide applicability in resonance-domain diffractive optics and in realization of quasi-planar metamaterials. We discuss design issues of such optical elements and demonstrate the method by fabricating narrow-band spectral filters based on the guided-mode resonance effect. These elements have strong potential for, e.g., sensing applications in harsh conditions.

## Introduction

In a variety of optoelectronic and photonic device applications, nanostructured optical elements are expected to withstand rather extreme operating conditions. Such (normally fragile) elements should also be reusable to be cost-effective in, e.g., sensing applications, especially if being a part of a complex integrated optical system. This requires that the surface can be cleaned without damage that would affect the optical functionality, which can be a major problem with bare surface-relief-type nanostructures. Ideally, therefore, one would wish the top surface of the nanostructure to be (at least nearly) flat.

In this paper we present a fabrication technique that satisfies the requirements stated above. We use Atomic Layer Deposition (ALD)^1–3^ to grow a thin film of Titanium Dioxide (TiO_2_) on top of a nanostructured surface-relief profile to form an element with a nearly flat top surface. TiO_2_ is an excellent choice as a cover material due to its transparency over a wide spectral bandwidth, stability at high temperatures, relatively large dielectric constant, and high mechanical and chemical robustness. When this technique is used, the optical function of the element is based on the internal nanostructure underneath the essentially flat surface. Hence the optical design of the elements involved differs from the design of bare surface-relief-type nanostructured elements; in general, the conformal nature of ALD growth needs to be considered.

We illustrate the fabrication process by applying it to a particular class of nanostructured elements, i.e., Guided-Mode Resonance Filters (GMRFs)^4,5^. These are narrow-band spectral filters based on guided-mode excitation anomalies in diffraction gratings with wavelength-scale periodicity, exhibiting strong reflection peaks at wavelengths that depend on the structural parameters of the grating. Such filters have received a great deal of attention over the past decades, and found extensive use in, e.g., sensing applications^6^.

The paper is structured as follows. We begin by describing the proposed fabrication method and associated design issues on a general level. The parametric design of buried GMRF structures by means of the Fourier Modal Method (FMM)^7^ is described in the following section. The fabrication process involving electron beam lithography, dry etching, and ALD is covered in the fabrication section. Experimental results are further described, and finally discussion and conclusions are drawn.

## Fabrication Method and General Design Issues

**Figure 1:**
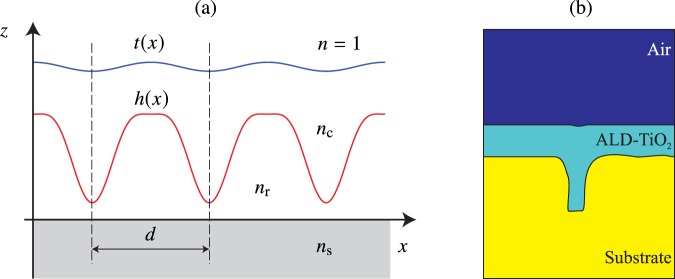
The ALD growth process. (**a**) Starting from a nanostructured surface profile (red line), material is grown conformally until a desired slightly modulated top profile (blue line) is obtained. (**b**) Simulation of a 50-nm-thick TiO_2_ layer grown by ALD. The substrate shape is the contour of a real grating extracted from an SEM picture. The video illustrates the conformal growth as a function of time, i.e., the number of deposition cycles (see the video in Supplementary document).

The principle of the proposed fabrication technique is illustrated in Fig. 1(a). Here a periodic initial surface profile *h*(*x*) of period *d* is shown, which is made of a material of refractive index *n*_r_ on a flat substrate of refractive index *n*_s_. Non-periodic and three-dimensionally modulated profiles can be used as well, but it is essential that the original surface profile is at least quasi-periodic with a local period of the order of the wavelength or less, as is usual in resonance-domain diffractive optics^8^. The profile *h*(*x*) is coated by conformal ALD growth of material of refractive index *n*_c_. This process can be stopped at any stage; indeed, the method has recently been used to coat diffractive surfaces with thin layers of high-index materials^9–13^. However, when the growth is continued, the grooves in the original nanostructure *h*(*x*) are ultimately filled and the modulation of the top surface becomes smoother. This is illustrated by simulation of conformal growth in Fig. 1(b). Finally the top surface becomes only weakly modulated and the result is the desired robust structure that can be easily cleaned.

The advantages of the proposed technique are not limited to achieving easily cleanable diffractive elements. In many cases resonance-domain and subwavelength-structured diffractive elements have high aspect ratios, i.e., the height of the modulated profile can be of the same order of magnitude or larger than the period. Such high-aspect-ratio structures tend to be very fragile, which is avoided by filling the nanostructure with a solid material. More generally, the use of ALD as a post-process re-coating technique is proven to be highly advantageous in micro- and nano-photonics^14^. Recent demonstrations have shown the potential of the technique to reduce the scattering loss in waveguides^15,16^, to decrease the effective index of integrated complex nanostructures^17^, and fine tune the geometrical parameters of tiny patterns to reach a precise response^18,19^. Concerning sub-wavelength gratings covered by atomic layer deposition, only one study has been published so far^20^.

There are some general design issues that must be considered when the proposed technique is used. The idea is to create a volume-type element, in which the internal structure *h*(*x*) primarily defines the optical function, whereas the top surface *t*(*x*) preferably provides only a negligible contribution to the diffraction characteristics. To achieve this, we need a high index contrast between *n*_r_ and *n*_c_, which is achieved, for example, by coating a SiO_2_ substrate with TiO_2_. However, since *t*(*x*) is a surface profile, one must at least check its effect in the optical performance of the element. Another issue, due to the high refractive index *n*_c_ of the coating material, is the reflection loss at the top surface. If necessary, this loss can be reduced by ALD growth of additional layers of dielectric materials with different refractive indices on top of the final profile *t*(*x*).

**Figure 2:**
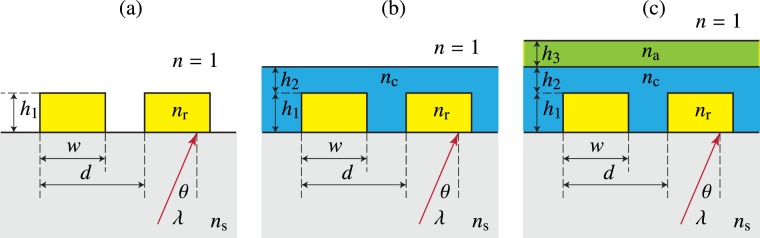
(**a**) A bare binary grating illuminated by a plane wave. (**b**) A binary grating buried completely by ALD growth of material with refractive index *n*_c_. (**c**) A buried grating coated with an additional ALD-grown layer of material with refractive index *n*_a_. We consider *n*_s_ ≈ *n*_r_ and *n*_c_ > *n*_*a*_ > *n*_*s*_ > *n*.

We proceed to discuss the above-mentioned issues with the aid of some examples on the design of buried binary diffraction gratings illustrated in Fig. 2. The bare grating structure shown in Fig. 2(a) is characterized by the period *d*, the ridge height *h*_1_, and the ridge width *w* (or the fill factor *f* = *w*/*d*), in addition to the refractive indices *n*_s_ and *n*_r_. We assume that the grating is illuminated from the substrate side by a plane wave at an angle of incidence *θ*. The buried grating in Fig. 2(b) is assumed (ideally) to have a flat top surface at a height *h*_2_ above the ridges of the underlying grating. Finally, in Fig. 2(c), we illustrate the option of adding a thin film (thickness *h*_3_) of material with refractive index *n*_a_ on top of the buried grating.

**Figure 3:**
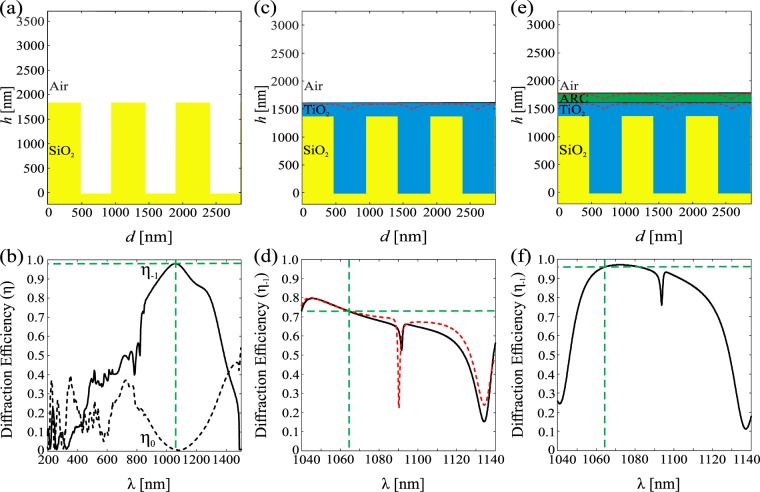
Structures and efficiency curves of optimized surface-relief Bragg gratings. (**a**) A bare binary grating made of SiO_2_ and (**b**) its efficiency curves *η*_−1_(*λ*) and *η*_0_(*λ*). (**c**) A binary SiO_2_ grating buried by ALD growth of TiO_2_ and (**d**) its efficiency curves. (**e**) A buried grating coated with an Al_2_O_3_ and **(f**) its efficiency curve. In (**c**,**e**) the black lines indicate the ideal profiles and the red dotted lines are simulations of ALD-grown structures. In (**d**) the black and red dotted lines are the efficiency curves for the ideal and ALD-grown structures, respectively. Note the wide horizontal scale in (**b**) compared to (**d**,**f**), where only the spectral region of main interest is covered.

In the forthcoming sections we will consider GMRFs in detail, but some of the design issues discussed above can be illustrated more clearly using Bragg-type surface-relief gratings as underlying structures. Such gratings have been used, e.g., as carrier gratings in coding complex optical signals in diffractive optics^21–23^. The optical function of a (regular transmission-type) Bragg grating is to diffract a maximum fraction *η*_−1_ of incident light into the first diffraction order *m* = −1, when illuminated at the Bragg angle *θ* = *θ*_B_ given by $\sin \,{\theta }_{{\rm{B}}}=\lambda \mathrm{/2}{n}_{{\rm{s}}}d$. A high diffraction efficiency *η*_−1_ is achieved by proper design of the parameters *w* and *h* if the grating period is chosen in such a way that only transmitted orders *m* = −1 and *m* = 0 (the zeroth order) can propagate, i.e., *d* < 3*λ*/2. Choosing, for example, *n*_s_ = *n*_r_ = 1.45 (SiO_2_) at a design wavelength *λ* = 1064 nm and apply the Fourier modal method as in Turunen *et al*.^21^, we obtain *η*_−1_ ≈ 97% with a design *d* = 958 nm, *w* = *d*/2, and *h* = 1851 nm. Figure 3(a) shows a scale drawing of the structure and Fig. 3(b) illustrates the efficiency curves of transmitted orders *m* = −1 and *m* = 0 of this grating. The efficiency curve is smooth around *λ* = 1064 nm, showing that the design is not exceedingly wavelength-sensitive. A smooth efficiency curve is obtained also if the wavelength is kept fixed but the angle of incidence is varied around the Bragg angle *θ*_B_.

Let us next assume an ideal (flat-topped) structure shown in Fig. 2(b) with a coating material of refractive index *n*_c_ ≈ 2.33 (TiO_2_). If we keep *d* and *w* at the same values as above and choose *h*_1_ = 1383 nm and *h*_2_ = 239 nm, we obtain an efficiency curve shown in black in Fig. 3(d). The efficiency curve of this design shows rapid fluctuations as a function of wavelength, and similar behavior is also observed if *λ* is kept fixed but the angle of incidence is varied. These fluctuations can be attributed to strong multiple scattering effects due to the high index contrast between the top layer of the structure and air, which lead to Fabry-Perot type resonances. To reduce such effects, we add of a lower-index top layer (Al_2_O_3_ with *n*_a_ ≈ 1.59)^24^ in a way schematically illustrated in Fig. 2(c). This layer is intended to act as an antireflection coating of the top surface, and since *n*_a_ is not too far from the ideal index-matching value $\sqrt{{n}_{{\rm{c}}}}\approx 1.53$ of a single-layer antireflection coating, we expect a quarter-wave layer of Al_2_O_3_ (thickness *h*_3_ = *λ*/4*n*_a_ ≈ 167 nm) to be a good estimate for the optimum thickness of such a layer. Indeed, keeping *d* and *w* at the same values as above and optimizing the thicknesses, we obtain a solution *h*_1_ = 1383 nm, *h*_2_ = 239 nm, *h*_3_ = 167 nm, which has a smooth efficiency curve around the design wavelength, shown in Fig. 3(f). The efficiency at *λ* = 1064 nm is *η*_−1_ = 95.9%, which is close to the value obtained for the bare-grating design.

As already mentioned, it is important to check the effect of the actual profile of ALD grown structures in the performance of the designed elements. In Fig. 3(d), we show simulations of ideal profile and ALD-grown structures at the stage where the target thicknesses specified above for idealized flat-top profiles are roughly achieved. Small dips on the top surface remain between the ridges, as expected in view of simulations in Fig. 1(b). However, the effect of these dips in the efficiency curves is almost negligible.

## Design of Buried Guided-Mode Resonance Filters

A binary grating of the type illustrated in Fig. 2(a) can act as a GMRF with a suitable choice of the parameters. If *n*_r_ is sufficiently large compared with *n*_s_, the grating acts as a leaky waveguide: resonant coupling of incident light then produces sharp reflection peaks at certain wavelengths that depend on the state of polarization and the angle of incidence of the illuminating wave, and on the choice of the grating parameters^4,5^.

In this paper we study GMRFs of the form illustrated in Fig. 2(b). Starting from a binary surface-relief grating, which is etched directly into the substrate (so that *n*_r_ = *n*_s_), we grow higher-index material on top of it by ALD to form the buried structure. The grating is then defined by four parameters *d*, *f* = *w*/*d*, *h*_1_, and *h*_2_. We choose these parameters in such a way that, at a given angle of incidence *θ*, a zero-diffraction-order reflection peak corresponding to the lowest guided mode of the leaky waveguide (in either TE- or TM-polarization) occurs at some specified wavelength and has a certain desired width that depends on the application. We note that the resonance wavelength can be fine tuned simply by changing the angle of incidence *θ*. We also note that the structure is illuminated, in this case, from the air-side, with a view to the sensing experiments conducted further in this article.

We used FMM to find optimal structural parameters for inorganic GMRFs buried with TiO_2_ cover layers. Two designs called GMRF-I and GMRF-II are considered. The structural parameters of GMRF-I are *d* = 310 nm, *w* = 250 nm (*f* = 0.81), *h*_1_ = 85 nm, and *h*_2_ = 120 nm. The parameters of GMRF-II are *d* = 310 nm, *w* = 225 nm (*f* = 0.73), *h*_1_ = 75 nm, and *h*_2_ = 95 nm. Both gratings were designed for the incidence angle *θ* = 18°. To test the applicability of these GMRFs in sensing applications, we assume that an additional thin film of material (to be sensed) is located on top of the buried grating, which effectively implies a structure in Fig. 2(c). Both GMRF-I and GMRF-II produce TE- and TM-resonances, the positions of which depend on the thickness *h*_3_ and refractive index *n*_a_ of the additional layer.

**Figure 4:**
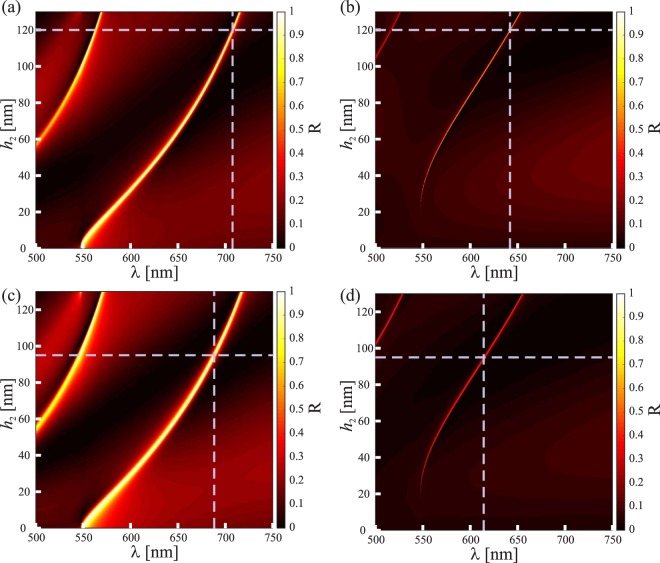
Simulated spectral variations as a function of increasing TiO_2_ thickness for GMRF-I (**a,b**) and GMRF-II (**c,d**) structures for TE (left) and TM (right) polarizations.

Unlike in the Bragg-grating example considered in the previous section, the addition of a layer of refractive index *n*_a_ and thickness *h*_3_ in Fig. 3(f) is not necessary to achieve the desired optical response of the GMRFs, though it could improve the transmission outside the guided-mode resonance. We use such a layer here for a different purpose: to introduce a material to be sensed. In the experiments we test several materials: Polymethyl Methacrylate (PMMA), Polyvinyl Alcohol (PVA), and AZ nLOF. Figure 4 illustrates the calculated spectral positions of the reflection resonances as a function of *h*_2_. We consider *n*_c_ to be the refractive index of TiO_2_, taking into account the dispersion of the material, and *n*_a_ = 1 (air). The zeroth-order reflectance is plotted here over a wavelength range *λ* = 500–750 nm with a step size of 0.1 nm. For small values of *h*_2_ only a single resonance peak is observed in both TE- and TM-polarization, but a second appears when *h*_2_ is increased sufficiently to allow excitation of a second guided mode in the GMRF structure. We are primarily interested in the spectral position of the fundamental-mode resonance.

## Fabrication

**Figure 5:**
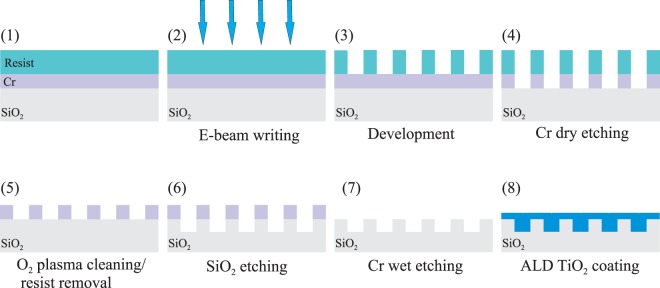
Fabrication process of ALD-TiO_2_ buried guided mode resonance filters (GMRFs).

The fabrication process of the GMRFs is illustrated in Fig. 5. We employed Electron Beam Lithography (EBL) for patterning, Reactive Ion Etching (RIE) to make the underlying grating profile in fused silica, and Atomic Layer Deposition (ALD) to bury the SiO_2_ grating by amorphous TiO_2_.

The first fabrication step is the creation of a chromium hard mask supporting the plasma etching of the silica. A 50-nm Cr layer is deposited on the wet cleaned substrate by electron beam evaporation (Lebold L560) on top of which a positive tone e-beam resist (AR-P 6200) is spin-coated. The patterns corresponding to the two GMRF designs are written in the resist by electron beam lithography (EBG5000 + ES HR from Vistec) under an acceleration voltage of 100 kV. The resist layer is developed using OPTIspin SST20 as chemicals and the pattern is transferred to the Cr layer by inductively coupled plasma reactive ion etching (ICP-RIE, Plasmalab 100, Oxford Plasma Technology) under Cl_2_ and O_2_ gases. The pattern is further transferred to the fused silica substrate by another inductively coupled plasma etching step using CHF_3_ and Ar as plasma gases (Plasmalab 80, Oxford Plasma Technology). The excess of Cr is removed by wet etching.

The final fabrication step is the coating of the grating by a TiO_2_ layer using ALD (TFS 200, Beneq). The quality and optical properties of this layers, that are crucial in our work, depend strongly on the precursors used in this process, i.e., TiCl_4_ and H_2_O, and on the process temperature and speed, which were chosen as *T* = 120 °C and 0.07 nm/cycle, respectively. With these parameters, the deposited TiO_2_ is amorphous and its refractive index at visible frequencies is around 2.4 (*λ* = 550 nm).

**Figure 6:**
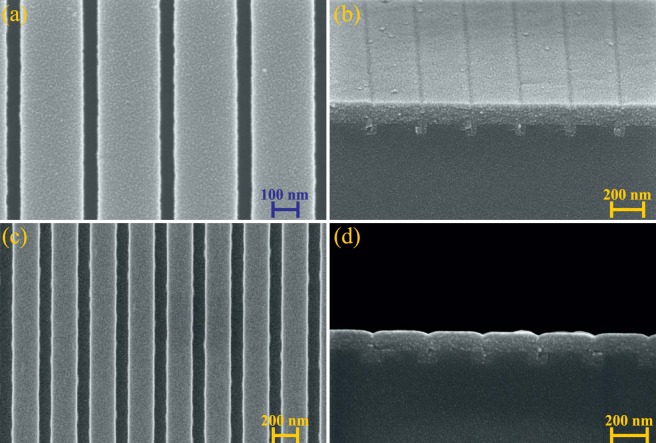
SEM pictures of the structures. (**a**,**c**) Show the top surfaces of GMRF-I and II, respectively, before the TiO_2_ deposition. (**b**,**d**) Are cross-sectional profile pictures of GMRF-I and II, respectively, after the TiO_2_ deposition.

Scanning Electron Microscopy (SEM) is used to evaluate the quality of the fabricated GMRFs. Figure 6(a,c) show the top surface of the silica grating before ALD-TiO_2_ coating. The period and the fill factor are very well respected, however a slight roughness appears on the sides of the grooves. Figure 6(b,d) are cross-sectional pictures of the structures after ALD-TiO_2_ coating. It is clear that ALD offers the clear advantage of conformal coating, filling perfectly the grooves and thus burying the gratings completely. However, as expected from Fig. 1, a weak modulation remains above the grooves. It is to be noted that these SEM pictures have been taken after all the measurements we will present in the rest of this article, including the influence of an additional polymer coating on top of the grating (see the following section). We remark that no traces of any materials are visible on top of the GMRFs, except the Cu layer used for imaging. This means that our concept of nearly flat-top buried grating is perfectly working as a robust structure that can be cleaned after use.

Optical profilometer (NanoCalc Thin Film Reflectrometry System, Ocean Optics, Inc.) and Variable Angle Spectroscopic Ellipsometer (VASE, J. A. Woollam Co.) measurements of test samples showed that the thickness of the ALD-TiO_2_ layers is within an uncertainty of ±5 nm compared to the targeted thicknesses. It is to be noted that such a small variation yields already a consequent shift of the resonance. According to FMM calculations, a variation of ~5 nm on the TiO_2_ thickness leads to a ~4 nm shift of the TE response, and about ~6 nm for TM. We therefore expect to see slight variations in the experiments compared to the theory.

## Optical Characterizations and Experimental Results

### Spectral measurements

**Figure 7:**
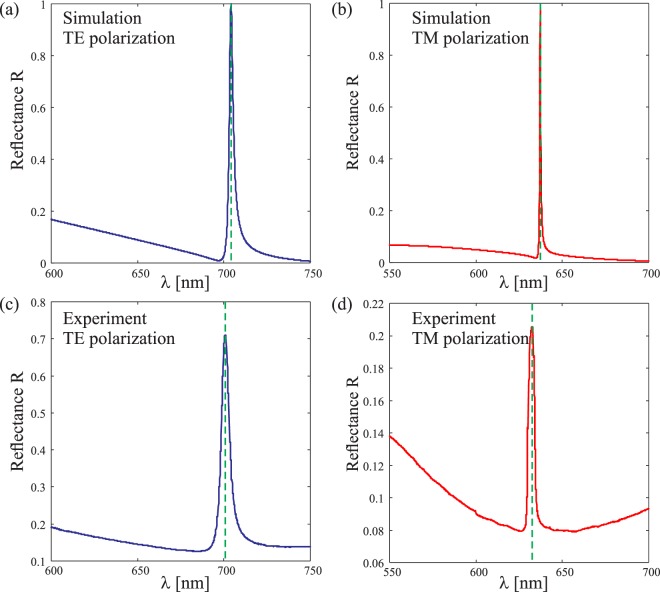
Simulated and experimental reflectance spectra of GMRF-I at incidence angle *θ* = 18° for: (**a**,**c**) TE- and (**b**,**d**) TM-polarized light.

**Figure 8:**
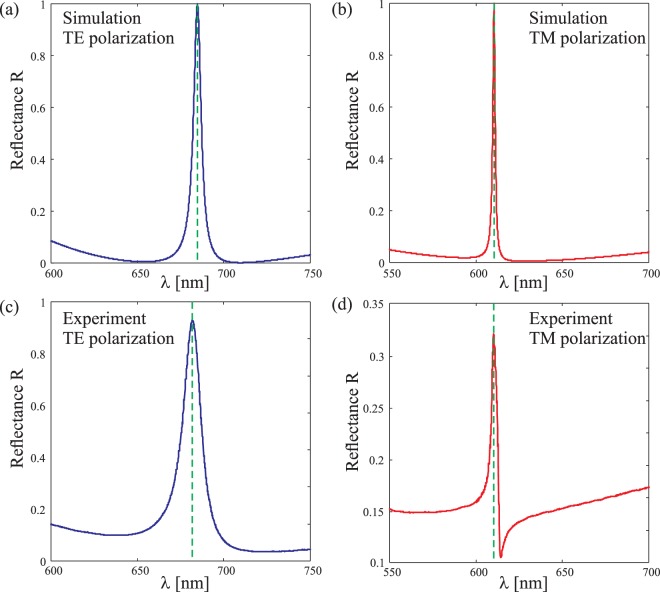
Simulated and experimental reflectance spectra of GMRF-II at incidence angle *θ* = 18° for: (**a**,**c**) TE- and (**b**,**d**) TM-polarized light.

Spectral measurements were performed in reflection using the above-mentioned ellipsometer over the wavelength range 550 nm ≤ *λ* ≤ 700 nm, with the incidence angle set at *θ* = 18°. Simulated and experimental TE- and TM-reflectance spectra for GMRF-I and GMRF-II are shown in Figs 7 and 8, respectively.

At first glance one can remark an excellent match between experiments and simulations for both gratings, apart from the fact that the peak experimental reflectance does not reach unity. This is particularly evident in TM-polarization, for which the theoretically predicted resonance linewidths are considerably narrower than they are in TE-polarization. The main reason is that, for narrow spectral resonances, a highly collimated beam illuminating a large area of the grating is required to observe perfect reflectance at the resonance, and this condition was not fully satisfied in our experiments. This, however, does not affect the spectral positions of the resonances significantly; it mainly reduces the peak reflectance of the grating and increases the effective width of the resonance lines.

A closer look at the results tells us that the spectral responses of the fabricated structures are a little shifted compared to the designed ones. As already explained, this was expected, and it does not affect sensing applications if the peak position is first determined experimentally for the structure without a sensing-material layer. The designed resonant wavelength peak position for TE-spectra of GMRF-I is *λ* = 703.9 nm for a TiO_2_ thickness *h*_2_ = 120 nm, while the measured position is *λ* = 700.8 nm. The full width half maximum (FWHM) of the TE-reflectance peak of GMRF-I is approximately 2.8 nm in design and 5.4 nm in experiments. The designed TM-reflectance peak position is *λ* = 633.8 nm (FWHM = 0.7 nm) and the experimental value is *λ* = 632.6 nm (FWHM = 4 nm) for GMRF-I. Similar variations can be seen between the designed and measured results for GMRF-II.

Shifts in the resonance peak position are expected to occur due to small variations of *h*_2_ from the design value, but other reasons also contribute to the spectral shifts and the shape variations of the resonances. First, as seen from the SEM pictures (Fig. 6), a weak modulation of the top surface remains after burial. This modulation is equivalent to a variation of the thickness or the refractive index of the cladding layer, which yields to a variation of the effective index of the guided modes. The influence on the thickness has already been discussed and a 1%-variation in the refractive index of TiO_2_ leads to a 5 nm shift of the resonance. Second, a slight misalignment of the sample in the ellipsometer is equivalent to a rotation of the polarization and yield again a shift of the resonance. One can see that between TE- and TM-polarizations, the resonance wavelength is set more than 50 nm apart. Finally, a small uncertainty in the illumination angle *θ* of only 1° yields a drift of about 4 nm of the spectrum. Clearly, any of the errors mentioned above have a far greater effect in TM polarization than in TE polarization because of the substantially narrower linewidth in the TM case. Apart from reduced peak intensity, these errors cause differences between the simulated and measured TM resonance line shapes, which is particularly evident in Fig. 8(b,d).

### Influence of the cladding medium

The main advantage of a buried resonant grating featuring a flat top surface is its robustness, which offers great additional value in sensing and bio-sensing, for instance. In order to verify this functionality of our device, we have performed a series of measurements with different top layers (above the TiO_2_ layer). Since we use an ellipsometer for measurements, forcing the sample to be in a vertical position, we used several polymers of different refractive indices and spin-coated on the grating to mimic a potential future analyte. Polymers we used are chosen to have different refractive indices in the visible region: we tested Polymethyl Methacrylate (PMMA, ${n}_{{\rm{a}}}\simeq 1.47$), Polyvinyl Alcohol (PVA, ${n}_{{\rm{a}}}\simeq 1.53$), and AZ nLOF 2070 (nLOF, ${n}_{{\rm{a}}}\simeq 1.63$). Some polymers, such as PVA, have a very low viscosity leading to very thin layers. We targeted to such thin layers in order to observe the behavior of our device when only the refractive index at the vicinity of its surface is changing. The speed of spin-coating was adjusted so that the thickness of the polymer layer is around *h*_3_ = 65 nm.

**Figure 9:**
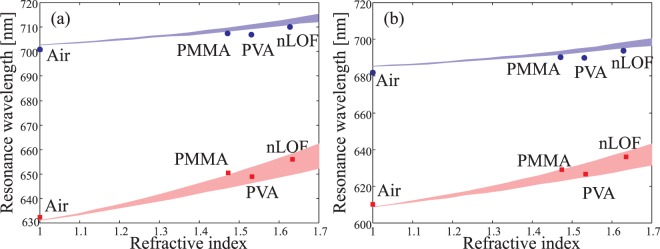
The experimental sensing results of three different polymer materials of thickness ${h}_{3}\simeq 65$ nm tested separately on the same (**a**) GMRF-I and (**b**) GMRF-II structures in both TE- and TM-polarization as a function of increasing sensing material refractive index. Blue and red colors correspond to TE- and TM-polarization respectively.

Simulation and experimental results are presented in Fig. 9 for TE- and TM-polarizations and for both grating designs. In this figure, we report the experimental data points measured with the above mentioned setup together with the simulations (colored areas). Calculations have been done for 50 nm ≤*h*_3_ ≤ 90 nm in order to consider the fluctuation due to the viscosity of the polymer and the rotation speed of spin coating. The colored areas in the theoretical predictions represent this range.

Apart from a slight variations between theory and experiments, the simulations predict the same trend as experiments show if we consider the uncertainty of *h*_3_. The gratings were cleaned between subsequent measurements, meaning that the devices survived the effects of different chemicals used to remove the polymers. PVA is dissolved in hot water at 90 °C, PMMA is removed by acetone in ultrasonic washer at 40 °C for 5 min, and nLOF is removed with AR 300-47 developer solution for 90 s followed by deionized water rinsing for 30 s. Before each new coating, measurements with no sensing layer were done to ascertain the reliability of the grating after use. A maximum average variation ${\rm{\Delta }}\lambda \simeq 1$ nm in resonance peak positions was observed, which proves that the structures can be reused even after harsh chemical and mechanical treatments.

From the results in Fig. 9, one can also extract the sensitivity of the device, i.e., the resonance wavelength shift Δ*λ*_R_ as a function of the refractive index variation Δ*n*_*a*_: *S* = Δ*λ*_R_/Δ*n*_*a*_^25^. The measured sensitivities are for GMRF-I, ${S}_{{\rm{TE}}}^{{\rm{I}}}=14$ nm/RIU and ${S}_{{\rm{TM}}}^{{\rm{I}}}=35$ nm/RIU for the TE- and TM-polarization, respectively. For GMRF-II, the measurement led to a sensitivity of ${S}_{{\rm{TE}}}^{{\rm{II}}}=17$ nm/RIU and ${S}_{{\rm{TM}}}^{{\rm{II}}}=38$ nm/RIU. One can note that the slightly higher sensitivity of the second configuration is explained by a thinner TiO_2_ layer, allowing a better overlap of the tail of the resonant mode compared to the first design. In both cases, the device is about twice more sensitive when illuminated under TM-polarization than under TE-polarization. Note that in a real sensing experiment involving polar molecules, the hydroxyl groups, inherent to the precursors used in the ALD process, at the surface of the layer, may yield a saturation of the sensor and limit its application. This can be avoided by adding a very thin buffer layer of SiO_2_ on top of the whole device. Such an additional layer will affect the optical response of the device, but can be taken into account in the design as mentioned in the design section of this paper^26^.

## Conclusions

We have proposed a fabrication technique, based on atomic layer deposition, to bury a nanostructured surface-corrugated profile of a resonance-domain diffractive element into a solid high-index material. This technique effectively transforms a surface-relief element into a volume-type element, but it retains the high refractive-index contrast necessary for efficient realization of the optical function of the element. While generally requiring a complete redesign of the nanostructure to achieve the desired optical functionality, the proposed method offers a way to overcome problems with fragileness and environmental sensitivity of high-aspect-ratio surface-corrugated diffractive nanostructures. We expect the method to find widespread use in diffractive optics and in realization of subwavelength-featured quasi-planar metamaterials. In particular, we applied the proposed techniques to design, fabricate, and characterize ALD-TiO_2_ buried guided-mode resonance filters, and tested these elements in sensing applications. Owing to the nearly flat top surface provided by the fabrication technique, we were easily able to clean the elements from the remainders of one sensing material before re-use with an another material, without any noticeable damage or deterioration of the optical response.

## Electronic supplementary material

Supplementary Fig. 1(b)
